# Post-Transcriptional Control of Chloroplast Gene Expression

**DOI:** 10.4137/grsb.s2080

**Published:** 2009-03-12

**Authors:** Eva M. del Campo

**Affiliations:** Department of Plant Biology, University of Alcalá, Alcalá de Henares, 28871-Madrid, Spain

**Keywords:** Chloroplasts, primary transcripts, RNA processing, RNA stabilization, intron splicing, RNA editing, PPR protein

## Abstract

Chloroplasts contain their own genome, organized as operons, which are generally transcribed as polycistronic transcriptional units. These primary transcripts are processed into smaller RNAs, which are further modified to produce functional RNAs. The RNA processing mechanisms remain largely unknown and represent an important step in the control of chloroplast gene expression. Such mechanisms include RNA cleavage of pre-existing RNAs, RNA stabilization, intron splicing, and RNA editing. Recently, several nuclear-encoded proteins that participate in diverse plastid RNA processing events have been characterised. Many of them seem to belong to the pentatricopeptide repeat (PPR) protein family that is implicated in many crucial functions including organelle biogenesis and plant development. This review will provide an overview of current knowledge of the post-transcriptional processing in chloroplasts.

## Introduction

**Figure 1:**
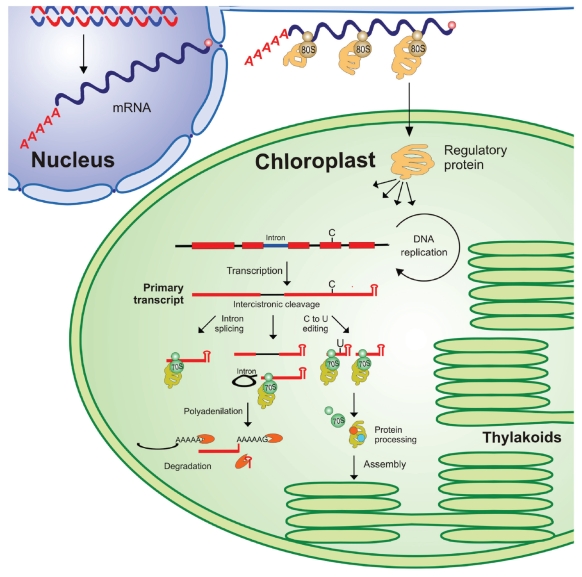
Schematic representation of the mechanisms involved in the control of chloroplast gene expression in higher plant chloroplasts Most of the genes encoded in higher plant chloroplasts are organized as operons.^9^,^10^ Primary transcripts are further modified to produce functional RNAs. In higher plants, post-transcriptional modifications include RNA cleavage of pre-existing RNAs, RNA stabilization, intron splicing and RNA editing. Generally, RNA editing affects mRNAs RNA stabilization usually involves the formation of a 3′ stem loop secondary structure which prevents its 3′ to 5′exonucleolytic degradation.^38^ Most of chloroplast introns in higher plants belong to group II and are spliced by releasing the intron in a lariat form.^105^,^106^ Generally, editing is found mRNAs but it also affects structural RNAs. In chloroplasts, most editing events involve conversions of cytidine (C) to uridine (U), but they are also “reverse” conversions of uridine to cytidine as is the case of several studied lower plants as hornworts and ferns.^120^–^123^ Several nuclear-encoded proteins participate in diverse plastid RNA processing events. Many of them seem to belong to the pentatricopeptide repeat (PPR) protein family that is implicated in many crucial functions including organelle biogenesis and plant development.^172^

Since the discovery of the existence of DNA^1^ and ribosomes^2^ in chloroplasts, many studies have been published about the structure of the chloroplast genome and its expression. These studies were facilitated by the development of cloning and sequencing techniques in the 1970s. The first physical map of plastid DNA was obtained from maize^3^ and the first plastid gene was cloned in 1977.^4^ A decade later, the complete chloroplast genome of tobacco,^5^ *Marchantia polymorpha*^6^ and rice^7^ was sequenced. These first approaches culminated in an emerging new field: gene organization and expression of the chloroplast genome. This field has subsequently become one of the most studied in plant molecular biology. The chloroplast genome has both prokaryotic and eukaryotic properties,^8^ but resembles prokaryotic systems since it has σ^70^ type promoters, a plastid encoded RNA polymerase, operons, “Shine-Dalgarno”-like sequences, and 70S ribosomes. The chloroplast genetic machinery also has characteristics of nuclear systems with the presence of introns and highly stable mRNAs. Consequently, the control of chloroplast gene expression includes several processes that are similar to those of prokaryotic and/or eukaryotic systems. These processes are: transcription, post-transcriptional processing, translation, and post-translational modifications. Generally, transcription rates and steady-state mRNA levels are not consistent suggesting that post-transcriptional RNA processing and stabilization are decisive steps in controlling plastid gene expression. This step principally includes RNA cleavage of pre-existing RNAs, RNA stabilization-degradation, intron splicing, and RNA editing (Fig. 1).

## Plastid Transcriptional Machinery

Most of the genes encoded in higher plant chloroplasts, including genes involved in related functions, are organized as operons. However, they may also encode functionally unrelated genes.^9^,^10^ Plastid operons are transcribed as polycistronic units by at least two distinct RNA polymerase activities: the plastid-encoded (PEP) and the nuclear-encoded (NEP) RNA polymerases.^11^,^12^ PEP is a multisubunit complex which resembles eubacterial RNA polymerases and is the predominant transcriptional activity in mature chloroplasts. PEP recognizes *E. coli* σ^70^ type promoters whose typical TTGACA (−35) and TATAAT (−10) consensus elements are found upstream of most plastid transcriptional units. The PEP core enzyme is composed of four different subunits, α, β, β′ and β,” which are encoded on the plastid genome by *rpoA*, *rpoB*, *rpoC1* and *C2* genes.^13^ The activity of the PEP core enzyme is regulated by sigma-like transcription factors (SLFs) that paly a role in promoter selection in a similar manner to the RNA polymerase of *E. coli.*^14^,^15^ Six different sigma factors, SIG1–SIG6, have been described for *Arabidopsis thaliana.*^16^ The mRNAs of these SLFs are translated in the cytoplasm and the corresponding proteins are subsequently imported as precursor proteins into the plastids. Recently, several investigations have elucidated the role of sigma factors by analyzing *Arabidopsis* T-DNA insertion lines with disrupted SIG genes. SIG2 is known to specifically transcribe some of the *tRNA* genes^17^ and the *psaJ* gene,^18^ SIG3 specifically transcribes the *psbN* gene in plastids,^19^ SIG4 is of specific importance for *ndhF* gene transcription,^20^ SIG5 has been shown to play an important role in the recognition of the blue-light dependent promoter of the *psbD* gene^21^ and SIG6 plays a more general role during early plastid differentiation and plant development.^22^

There is a second nuclear-encoded transcription activity in chloroplasts (NEP, nuclear-encoded plastid RNA polymerase) supplementary to PEP.^11^ Most NEP promoters have a core sequence motif YRTA and are known as type-Ia, similar to plant mitochondria promoters.^23^ A subclass of NEP promoters, known as type-Ib, shares a GAA-box motif upstream of the YRTA-motif.^24^ Type-II NEP promoters lack these motifs and possess crucial sequences located downstream of the transcription initiation site, represented by dicot *clpP* promoters.^25^ Unlike PEP, NEP is a single subunit enzyme sharing homology with the RNA polymerases of phage T3 and T7.^26^,^27^ Initially, a gene encoding NEP was sequenced in several plants.^26^,^28^,^29^ Further isolation of functionally distinct NEP activities in spinach chloroplasts^27^ and the identification of two genes for NEP-like isozymes in *Arabidopsis*^30^ suggested the existence of additional NEP activities.

Recent evidence indicates that NEP is represented by two phage-type RNA polymerases (RpoTp and RpoTmp) that have overlapping as well as gene-specific functions in the transcription of plastidial genes in *A. thaliana*. RpoTp is localized in chloroplasts whereas RpoTmp, exclusively found in dicots, is presumably localized in both mitochondria and chloroplasts. *In vitro* transcription assays revealed no significant promoter specificity for RpoTmp and the accurate transcription initiation from overlapping subsets of mitochondrial and plastidial promoters without the aid of protein cofactors.^31^ RpoTp is a catalytic subunit of NEP involved in recognition of a distinct subset of type I NEP promoters.^32^ Mutational approaches indicated that the plastid RpoTp RNA Polymerase is required for chloroplast biogenesis and mesophyll cell proliferation in *Arabidopsis.*^33^ Evidence indicates hat RpoTmp and RpoTp are involved in similar developmental events and that they are partially redundant.^33^,^34^ However, in contrast to the role assigned to RpoTp in both early and late stages of vegetative development in *Arabidopsis*, RpoTmp is required in early seedling development. It has been shown that RPOTmp fulfills a specific function in the transcription of the rrn operon in proplasts/amyloplasts during seed imbibition/germination.^35^ In chloroplast, RpoTp is tightly associated with thylakoid membranes and interacts with a RING-H2 protein that in turn mediates intraplastidial trafficking of the RPOTmp RNA polymerase.^36^ The same research work presented a model in which light determines membrane association and functional switching of RPOTmp by triggering the synthesis of the RING protein. Interestingly, comparison of plastidial promoters from tobacco and *Arabidopsis* revealed a high diversity, which may also apply to other plants.^37^ The diversity in individual promoter usage in different plants suggests that there are species-specific ways of controlling gene expression in plastids.

## Chloroplast RNA Processing and Stability

Evidence indicates that the control of chloroplast gene expression relies more on RNA processing and stability than on transcriptional regulation.^38^,^39^ In chloroplasts, polycistronic primary RNAs transcribed by PEP and/or NEP are generally processed into smaller transcripts which are further modified. RNA processing mechanisms remain largely unknown and sometimes controversial in spite of the diverse studies, focusing on several aspects of chloroplast gene expressionreviewed in:^40^,^41^ Nowadays, the fact that post-transcriptional RNA processing of primary transcripts represents an important step in the control of chloroplast gene expression appears to be well accepted.^42^,^43^ In several cases, alternative processing of polycistronic primary transcripts may cause the simultaneous stabilization and degradation of alternative transcripts, resulting in the enhancement and inhibition of their translation, respectively.^44^–^47^ Whether or not transcript processing influences its translation into proteins remains controversial. In light of this, several investigations indicated that intercistronic processing is crucial for the translation of chloroplast operons and that the translation of monocistronic forms is more effective than translation of polycistronic forms.^45^,^48^,^49^ Nevertheless, in some cases it seems that translatable transcripts can be produced by both direct transcription from the promoter and intercistronic cleavage of pre-existing transcripts.^9^,^48^ Additionally, recent investigations with transgenic lines have demonstrated that processing into monocistrons is not required for over-expression of transgenes and that they are efficiently translated.^50^ Unlike higher plants, in the green alga *Chlamydomonas reinhardtii*, translation seems to be an essential step in the regulation of chloroplast gene expression.^51^,^52^ In these algae, transcript processing is less important in controlling plastid gene expression than in higher plants since nearly all genes appear to be transcribed as monocistronic RNAs.

In chloroplasts, transcript stability is mainly influenced by the presence of 5′ untranslated regions (5′-UTRs) and 3′-UTRs, that seem to be necessary to prevent the rapid degradation or low accumulation of primary transcripts.^53^–^57^ Deleting or mutating them destabilizes the RNA, leading to reduced transcript accumulation and translation.^53^,^58^,^59^ Most of the plastid transcripts have short inverted repeat sequences (IR) that can potentially form a stem loop secondary structure (Fig. 1). In prokaryotic organisms, similar structures appear to play a crucial role in transcription termination of RNAs. However, in chloroplasts, transcription termination is very inefficient, resulting in considerable read-through transcription of downstream sequences.^50^,^60^,^61^ Therefore, the role of plastid 3′-UTRs differs from the role of its bacterial counterparts since they are more involved in transcript stability preventing 3′ to 5′ exonucleolytic degradation of transcripts than in the effective termination of transcription.^38^

Another post-transcriptional modification affecting transcript stability is RNA polyadenylation (Fig. 1). In chloroplasts, poly(A) tails are found in degradation intermediate 3′-ends that contain not only adenosine but also other residues, principally guanosine.^62^ In chloroplast extracts, polyadenylated RNAs are degraded faster than nonadenylated RNAs and are more abundant *in vivo* under specific conditions that promote RNA degradation. Thus, polyadenylation might promote plastid RNA turnover *in vivo* by targeting endonucleolytic cleavage products for degradation^63^–^66^ as described for bacteria^67^–^70^ and plant mitochondria.^71^,^72^ The molecular mechanism of RNA degradation in chloroplasts appears very similar to that of bacteria.^63^,^64^,^73^ The first step consists of endonucleolytic cleavage of the RNA molecule, followed by polyadenylation.^74^,^75^ The polyadenylated cleavage products, including mRNAs^63^,^64^,^73^ and released introns,^76^ are then directed to rapid exonucleolytic degradation by PNPase and possibly other exoribonucleases (Fig. 1).^65^,^74^ Recent studies have revealed that although this enzyme is essential for efficient 3′-end processing of mRNAs, it is insufficient to mediate transcript degradation revealing an additional function of this exoribonuclease in tRNA degradation in *Arabidopsis thaliana.*^77^

In the last few years, several nuclear-encoded proteins that participate in chloroplast transcript processing and stabilization have been characterised. Most of them have been studied in *Arabidopsis* mutants (see Table 1). CRS2 is a protein that is involved in the intercistronic processing of *rps7-ndhB* transcripts.^78^ Such RNA processing seems to be essential for *ndhB* translation. This protein was first described in *Arabidopsis* mutants known as “chlororespiratory reduction mutants,” with reduced chloroplast NDH activity. *crr2-1* and *crr2-2* are recessive mutant alleles responsible for the impaired accumulation of the NDH complex. HCF152, encoded by the gene *hcf152*, is a RNA-binding protein that is involved in the processing or stabilization of the *petB* transcripts within the *psbB-psbT-psbH-petB-petD* operon.^79^ This gene was first identified in the nonphotosynthetic mutant of *Arabidopsis* hcf152 which does not produce the cytochrome b6f. The P67 protein seems to participate in the processing and translation of specific chloroplast mRNAs in radish and *Arabidopsis*^80^ and PGR3 is a nuclear-encoded protein which might have different functions in conferring RNA stability to the primary tricistronic transcript of the *petL* operon.^81^ This regulatory protein was described in *pgr3* (proton gradient regulation 3) mutants of *Arabidopsis*, which display high chlorophyll fluorescence (HCF) because of a reduced level of the cytochrome b6/f complex.

Mutants of several plant species other than *Arqabidopsis* have revealed the existence of new nuclear-encoded proteins which participate in chlroplast RNA processing and/or stabilization. In maize, the CRP1 protein is required for the translation of the chloroplast *petA* and *petD* transcripts and for the processing of the *petD* mRNA from a polycistronic precursor.^82^,^83^ Analysis of double mutants that lack both chloroplast ribosomes and CRP1 function suggested that CRP1 activates a site-specific endoribonuclease independently of any role it plays in translation.

*Zmppr5* is the maize ortholog of the embyoessential *Arabidopsis* gene *Atppr5*. The protein product of this gene is bound *in vivo* to the unspliced precursor of *trnG-*UCC RNA.^84^ Null and hypomorphic *Zmppr5* insertion mutants are embryo viable but show deficiency in chloroplast ribosomes and die as seedlings. In these mutants, transcription of *trnG*-UCC is unaffected but their encoded transcripts are dramatically decreased. This observation, in addition to biochemical data, indicates that PPR5 stabilizes the *trnG*-UCC precursor by direct binding and protection of an endonuclease-sensitive site.

In the moss *Physcomitrella patens*, the *ppr531-11*-disrupted mutants display a significantly smaller protonemal colony, different chloroplast morphology, incomplete thylakoid membrane formation and a reduction of the quantum yield of photosystem II.^85^ Several analyses have demonstrated that PPR531-11 has a role in intergenic RNA cleavage between *clpP* and 5′-*rps12* and in the splicing of *clpP* pre-mRNA affecting the steady-state level of ClpP, which regulates the formation and maintenance of thylakoid membranes in chloroplasts.

In the unicellular alga *Chlamydomonas reinhardtii*, expression of the chloroplast *petA* gene-encoding cytochrome f, depends on two specific nucleus-encoded factors: MCA1, required for stable accumulation of the *petA* transcript, and TCA1, required for its translation.^86^ Mutants with tagged versions of MCA1 and TCA1 have low amounts of MCA1 or TCA1, show limited *petA* mRNA accumulation and cytochrome f translation, respectively. It has been proposed that a rapid drop in MCA1 exhausts the pool of *petA* transcripts, and the progressive loss of TCA1 further prevents translation of cytochrome f where de novo biogenesis of cytochrome b(6)f complexes is not required.

## Intron Splicing

Several chloroplast genes, encoding both structural RNAs and proteins, are interrupted by introns. In chloroplasts, introns are classified into two main groups according to their conserved primary and secondary structures as well as their different splicing pathways, these are termed group I and group II introns. Land plant chloroplast genomes contain c.a. 20 group II introns and a single group I intron (within the *trnL*-UAA gene). However, a relatively high number of group I introns have been reported for green algae within organellar LSU rDNAs.^87^–^89^ Group I introns are found more frequently in eukaryotes than in prokaryotes.^90^ Approximately 90% of all group I introns identified to date are found in fungi, plants, and algae. In organellar DNAs, group I introns are found in genes encoding rRNAs, tRNAs, and proteins but they are limited to genes encoding rRNAs in the nucleus. Group I introns are located in functionally vital loci and they must be removed from transcripts by splicing, a process which occurs co-ordinately with ligation of RNA exons.^91^,^92^ The intron folds to form a secondary structure consisting of ten domains, P1 to P10, each with specific roles in the formation of a catalytic core responsible for carrying out the splicing and ligation.^91^,^93^ Most of the conserved nucleotides correspond to the four short sequences P, Q, R, and S. These sequences are located in the same 5′ to 3′ order at variable distances from each other (form c.a. 20 nt to many hundreds). All of the group I introns, from several genetic systems of diverse organisms identified to date including green algae chloroplasts, have a U at their 5′-end and a G at their 3′-end.^91^,^93^ Splicing proceeds through two transesterification reactions^93^ with the first reaction involving cleavage at the 5′ splice site and simultaneous addition of guanosine to the 5′ intron end. The second reaction involves cleavage at the 3′ splice site with concomitant ligation of exons. Group I intron splicing may be autocatalytic (self-splicing) or maturase facilitated. Several proteins from fungal mitochondria encoded by group I introns promote their splicing *in vivo.*^94^ However, self-splicing has only been tested by an *in vitro* assay in mitochondrial groupI introns from *Aspergillus nidulans.*^95^ In *Chlamydomonas reinhardtii* it has been demonstrated the existence of nuclear genes that promote splicing of group I introns in the chloroplast 23S rRNA and *psbA* genes.^96^

A remarkable feature of group I introns is their ability to colonize new insertion sites resulting in their spread.^90^ Intron insertion can occur via two alternative processes: reverse splicing and intron homing. Reverse splicing involves the insertion of a free intron into the RNA and has been observed in mobile group I introns integrated into the small subunit rRNA of bacteria and yeast.^97^,^98^ Intron homing is the insertion of an intron into a homologous position within an intronless copy of DNA.^99^

Intron homing is catalyzed by endonucleases, and are called homing endonucleases (HEs). Encoded by open reading frames (ORF) within introns, they recognize and cleave the target gene. In eukaryotes, HEs are found within nuclear and organellar genomes including both mitochondria and chloroplasts. HEs comprise four families known as: LAGLIDADG, GIY-YIG, His-Cys box, and HNH.^99^,^100^ In chloroplasts, HEs belonging to the LAGLIDADG, GIY-YIG, and HNH families have been discovered. The most studied chloroplast HEs were found within green algae of the genus *Chlamydomonas*. The LAGLIDADG family includes I-CreI and I-Ceu-I proteins from the chloroplasts of C. *reinhardtii* and *C. eugametos* respectively which have only one LAGLIDADG sequence motif and function as homodimers. X-ray crystallography has generated structural models for group I intron-encoded I-CreI HE [23S rRNA gene from *Chlamydomonas reinhardtii* chloroplast].^101^

The LAGLIDADG motifs form structurally conserved alpha-helices packed at the center of the interdomain. The DNA-binding interface forms a four-stranded beta-sheet located on either side of the LAGLIDADG alpha-helices. The last acidic residue of the LAGLIDADG motif participates in DNA cleavage by phosphodiester hydrolysis.^100^ The GIY-YIG family includes monomeric enzymes which are characterized by the conserved GIY-(X_10–11_)-YIG motif. In chloroplasts, the ORFs in introns 2 and 3 (Cr.psbA2 and Cr.psbA3) within the *psbA* gene of *C. reinhardtii* contain variants of the GIY-YIG motif.^102^ The I-CreII protein is an ORF within intron 4 (Cr.psbA4) of the *psbA* gene of *C. reinhardtii*. This HE contains an H–N–H and possibly a GIY–YIG motif.^103^ This protein is a double-strand-specific endonuclease that cleaves fused *psbA* exon 4–exon 5 DNA. Cleavage of heterologous *psbA* DNAs has been demonstated indicating that the enzyme can tolerate multiple, but not all, substitutions in the recognition site.

Group II introns are broadly distributed in diverse genetic systems including the chloroplast genome. This intron group can be distinguished by its folding into a characteristic secondary structure consisting on six helical domains radiating from a central core. There are two exon binding sites (EBS1 and ENS2) located within domain I. These exon binding sites interact with two intron binding sites (IBS1 and IBS2) located within the first twelve nucleotides of the intron 5′ end^104^ and their splicing proceeds via two alternative pathways known as the “branching” and “hydrolytic” pathways. The branching pathway consists of two consecutive transesterification reactions. During the first reaction, the first nucleotide of the intron 5′ end establishes a temporary 2′–5′ bond with a bulging adenosine located within domain VI. After intron splicing, the 5′ and 3′ exons join and the intron is released in a lariat form. The alternative splicing pathway starts by the hydrolytic cleavage of the 5′-splice site instead of transesterification.^105^ In chloroplasts, most group II introns have a bulging adenosine within their domain VI and the splicing seems to occur via the branching pathway except for the *trnV*(UAC) transcripts.^106^ In spite of the fact that plastid group II introns are large ribozymes, since they seem to be auto-spliced *in vitro*, experimental evidence indicates that proteins are required for the efficient splicing of many group II introns *in vivo*, but to date, few group II intron splicing factors have been identified. Some of the protein factors are encoded within certain plastid group II introns, which contain genes for maturase-like proteins involved in their own splicing as well as of other intron-containing plastid genes^107^,^108^ whereas others are nuclear encoded. Several nucleus-encoded proteins necessary for the splicing of various subsets of the c.a. 20 chloroplast group II introns in vascular plants have been reported. CRS1 is one of the first to be characterized and is necessary for the splicing of the group II intron in the chloroplast *atpF* gene.^109^,^110^ Further investigations have demonstrated the participation of additional proteins in *atpF* intron splicing. One such proteins is the ZmWHY1 that co-immunoprecipitates with CRS1. ZmWHY1 is the maize ortholog of WHY1 which acts as nuclear the transcription factors involved in pathogen-induced transcription in potato and *Arabidopsis* (StWHY1 and AtWHY1 respectively). Genome-wide co-immunoprecipitation assays have shown that ZmWHY1 in chloroplast extract is associated with DNA from throughout the plastid genome and with a subset of plastid RNAs that includes *atpF* transcripts.

**Figure 2:**
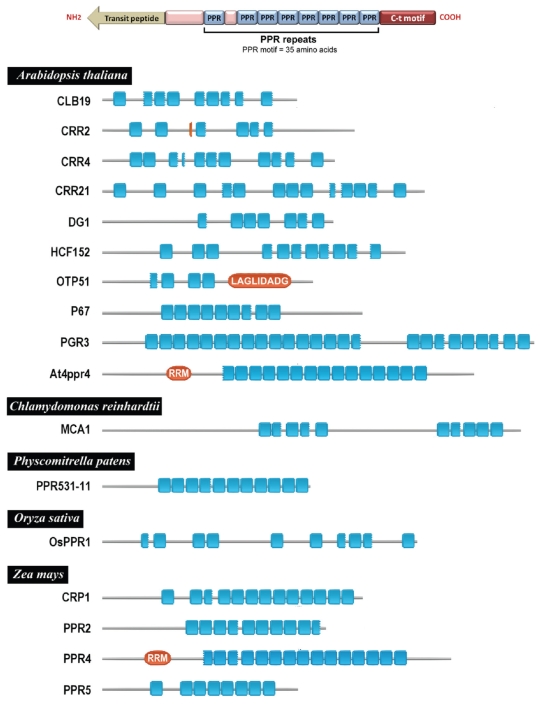
**(A) Structure of a hypothetical pentatricopeptide repeat protein. (B) Diagram depicting the PPR proteins listed in **Table 1 showing the distribution of PPR motifs. PPR motifs are represented as blue boxes whereas other motifs are represented as red ovals (RRM: RNA recognition motif; LAGLIDADG: LAGLIDADG motif). Only the motifs identified by using Pfam v21.10^183^ were represented. All depicted PPR proteins have a transit peptide (no represented) at their N-termini for their targeting to chloroplasts.

Various genetic approaches allowed the identification of additional nucleus-encoded proteins that are required for the splicing of group II introns in maize (*Zea mays*) chloroplasts: a CAF1/CRS2 complex, a CAF2/CRS2 complex, PPR4 and RNC1. Each of the afore mentioned nuclear-encoded factors is required for the splicing of distinct, but overlapping, subsets of the 17 group II introns in maize chloroplasts.^83^,^109^,^111^–^113^ CRS1, CAF1and CAF2 harbor a CRM domain which is a RNA binding domain^111^,^112^,^114^ and their *Arabidopsis* thaliana orthologs conserve the splicing functions.^110^ CRS2 is related to peptidyl-tRNA hydrolase enzymes^115^–^116^ whereas PPR4 is a member of the pentatricopeptide repeat (PPR) family (see Table 1 and Fig. 2).^113^,^117^ RNC1 is a plant-specific protein that has been recovered in both CAF1 and CAF2 co-immunoprecipitates^118^ and has two ribonuclease III (RNase III) domains. RNC1 is found in complexes containing a subset of group II introns in the chloroplasts that include, but are not limited to, CAF1- and CAF2-dependent introns. rnc1 mutants exhibit an inefficient splicing of many of the introns which are associated with RNC1 indicating that RNC1 promotes intron splicing *in vivo*. Despite its two RNase III domains, phylogenetic considerations and biochemical assays indicate that RNC1 lacks endonucleolytic activity. These and other results suggest that RNC1 promotes splicing via its RNA binding activity and that it is recruited to specific plastid introns via protein–protein interactions.

All of the investigations on nuclear-encoded splicing factors mentioned have contributed to the elucidation of the possible mechanisms by which they promote splicing. Nevertheless, the fate of introns after splicing remains an unresolved question. To this end, the analysis of the degradation products of *ndhA*, *atpF*, and *petB* transcripts in several plant species have demonstrated the existence of both incomplete introns and unspliced pre-mRNAs, which presumably correspond with their respective intermediate degradation products.^76^ Nucleotide sequencing of both 5′ and 3′ ends of such RNA species has shown that the cleavage affects specific intron domains and occurs within an unpaired bubble flanked by two-stem structures typical of prokaryotic RNAse III processing sites. Degradation of both unspliced pre-mRNAs and lariat introns has also been proposed as an additional mechanism that controls the level of mature translatable mRNAs of chloroplast genes.

## RNA Editing

In plants, with the exception of liverworts, RNA editing has been found in both mitochondria and chloroplasts.^119^ Generally, this post-transcriptional modification affects mRNAs but it can also affect structural RNAs. In chloroplasts, most editing events involve conversions of cytidine (C) to uridine (U), but “reverse” conversions of uridine to cytidine have also been noted in several studied hornworts and ferns.^120^–^123^ In the chloroplast of seed plants, about 30 different C to U transitions affecting mRNAs have been found.^124^–^128^ In bryophytes, the number of RNA editing sites in plastids range from zero in liverworts to almost 1,000 in hornworts.^121^,^122^,^129^ Editing often alters the amino acid identity and affects the amino acids that play a role in proper protein function.^122^,^129^,^130^–^132^ In some cases, editing creates new translation initiation codons, converting mRNAs into translatable messages or stop codons.^122^,^129^,^133^–^136^ The existence of these cryptic start codons created by RNA editing, led to the definition of open reading frames (ORFs). Editing sites have also been detected in the anticodon of tRNA (Leu) and within untranslated regions, including introns.^122^,^137^–^139^ However, it seems that the frequency of editing within non-coding regions is very low in comparison with the extent of editing within coding regions. The discovery that editing often leads to the conservation of certain amino acid residues in some proteins in both mitochondria and chloroplasts suggests that editing may act as a mechanism to prevent the deleterious effects of point mutations that have been maintained through evolution. The correspondence of 53 editing sites found in the fern *Adiantum capillusveneris* to editing sites in hornworts, and some other land plants, suggests that a major component of RNA editing sites have been conserved for hundreds of millions of years.^122^ Editing has also been studied in transcript processing intermediates to elucidate possible connections between editing and other post-transcriptional processing events (Fig. 1). The first results indicate that editing is an early RNA processing step, which precedes splicing and cleavage of polycistronic transcripts.^46^,^127^,^140^–^142^ The complete editing of polycistronic transcripts before any processing event could prevent aberrant forms of the corresponding protein as a result of the translation of unedited transcripts. The editing process involves two consecutive events: site recognition and nucleotide modification. It seems that the modification process in C to U transitions occurs via deamination of the base in plant mitochondria^143^,^144^ although the factor(s) mediating this process in plant organelles have not yet been identified. The recognition process for both mitochondrial and chloroplast RNA editing remains unknown to date. In this line, it is very difficult to explain the extraordinary high specificity in the selection of bases to be edited. Several studies indicate that RNA flanking sequences or cis-elements typically located within 15–30 nucleotides are involved in editing site recognition.^145^–^152^

Computational analysis of the sequences within −30 to +10 nucleotides of RNA editing sites (neighbor sequences) within the genomic and cDNA sequences of chloroplast genes in the moss *Takakia lepidozioides*. allowed statistical analyses of chloroplast RNA editing sites to be performed.^152^ This study allowed the development of a prediction algorithm wich predicted c.a. 60% of true editing sites in *T. lepidozioides* transcripts. The success of this prediction algorithm suggests that the obtained patterns are indicative of key sites recognized by trans-factors around editing sites of *T. lepidozioides* chloroplast genes.

One of the latest identified editing cis-elements is the 5′ sequence GCCGUU, which is required for editing of tobacco *psbE* transcripts *in vitro.*^153^ The analysis of *psbE* sequences from many plant species revealed that the GCCGUU sequence is present at a high frequency in plants that carry the same editing event of *psbE* transcripts with the exception of *Sciadopitys verticillata* (*Pinophyta*). This plant species showed editing at this site despite the presence of nucleotides that differ from the conserved cis-element. Interestingly, chloroplast extracts from a species that has a difference in the motif and lacks the C target are incapable of editing tobacco *psbE* substrates, indicating that the necessary trans-acting factors were not retained without a C target. Conversely, several heterologous editing events have been reported in different plant species indicating the maintenance of plastid RNA editing activities independently of their target sites.^154^

The transformation of the tobacco chloroplast genome has been extensively used to characterize *cis*-elements involved in editing site recognition in chloroplasts. These experiments, in combination with the introduction of point mutations, are very useful for identifying critical nucleotides that are targets for the editing apparatus.^146^,^147^,^155^ By using these techniques many cis-acting elements required for the editing process have been discovered. Evidence indicate that they are not specific to an individual editing site allow recognition of a cluster of editing sites even in transcripts of different genes.^156^ This finding is supported by the discovery of cis-elements of some 2–5 editing site clusters within different transcripts of various genes. Although lacking consensus sequences, they show some motifs in the 5′ region that are adjacent to editing sites that seem to be recognized by the same trans-elements.^157^ Moreover, it has been shown that editing sites that share trans-elements are edited to an equal extent under similar physiological contexts.^158^ In fact, in tobacco 34 editing events can be grouped into clusters of 2–5 editing sites according to sequence similarities immediately 5′ of the edited C. Analysis of transgenic tobacco plants with an over-expression of transcripts including each of the clusters showed impaired editing at these sites suggesting that the trans-factors are common to these editing sites therefore act as a limiting factor.^158^–^160^ Moreover, the expression of transgenes bearing the sequences surrounding an specific editing site in sense and/or antisense orientation affected editing efficiency of both transgenic and endogenous transcripts.^161^

Nowadays, scant data exist on the trans-factors responsible for this recognition. Various indirect data indicate that each editing site, or in some cases a small set of sites, must be recognized by specif ic factors encoded in the plant nuclear genome since it seems that editing is not dependent on the chloroplast translational apparatus.^162^,^163^ Nevertheless, recent studies on the influence of some physiological processes on editing revealed that treatments with antibiotics that inhibit translation in prokaryotes prevented certain C to U transitions.^164^,^165^ Recently, several nuclear-encoded proteins have been identified as possible trans-acting factors essential for RNA editing (see Table 1).^148^,^166^,^168^ CP31 is a RNA-binding protein required for the editing of two different tobacco sites *in vitro.*^148^ CRR4 and CRR21 are PPR (Pentatricopeptide) proteins essential for editing of a specific site in the chloroplast *ndhD* mRNA of *Arabidopsis thaliana.*^166^–^168^ Both CRR4 and CRR21 belong to the E+ subgroup of the PLS subfamily that is characterized by the presence of a conserved C-terminal region (the E/E+ domain). This E/E+ domain is highly conserved and exchangeable between CRR21 and CRR4, although it is not essential for RNA binding. It is possible that the E/E+ domain may have a common function in RNA editing rather than recognizing specific RNA sequences. CLB19 is a PPR protein similar to the editing specificity factors CRR4 and CRR21, but, unlike them, is implicated in the editing of two distinct target sites within the chloroplast, namely *rpoA* and *clpP* transcripts.^169^ Further studies will be necessary to characterize the entire editing machinery.

## The Role of PPR Protein in the Control of Chloroplast Gene Expression

A high number of nuclear mutants with non-photosynthetic phenotypes showing alterations in post-transcriptional steps have been isolated in higher plants^170^,^171^ and in the green alga *Chlamydomonas reinhardtii.*^43^ Generally, these mutants are affected in a single gene cluster or RNA. However, in some cases a single nuclear mutation simultaneously affects posttranscriptional processing of various operons.^48^,^109^ The existence of these mutants suggests, on one hand, the existence of nuclear-encoded factors that control chloroplast RNA processing, and on the other hand, that such processing could play a crucial role in controlling chloroplast gene expression. Recently, several nuclear-encoded proteins that participate in chloroplast transcript processing and stabilization have been characterised (see Table 1). Many of them seem to be pentatricopeptide repeat (PPR) proteins which are implicated in many crucial functions including organelle biogenesis and plant development.^172^

The PPR protein family is characterized by a degenerate motif (PPR motif) consisting of around 35 amino acids that occurs in multiple tandem copies (Fig. 2).^173^ The structure of these proteins is similar to other proteins with a repeat motif known as the tetratricopeptide repeat (TPR) involved in protein-to-protein interactions.^174^ Both TRP and PPR proteins share several structural similarities: i) they have degenerate helical tandem repeat motifs, TPR and PPR, respectively; ii) these repeat units form a super helix to bind biomolecules; iii) each repeat consists of anti parallel alpha helix (A and B); and, iv) they have conserved tyrosine residues that facilitate intra helix packing. In spite of these similarities, significant differences between TPR and PPR proteins remain: i) PPR proteins are predominant in plants and generally absent in prokaryotes, whereas TPR proteins are mostly abundant in animals and lower plants and present in prokaryotes; ii) PPR proteins interact with nucleic acids binding to a single target molecule (mainly single stranded RNA) whereas TPR interacts mainly with other proteins and may bind to multiple target proteins forming a complex; ii) PPR proteins have a higher number of repeats (2 to 27) than TPR proteins (3 to 16); iii) they have repeat units of 35 and 34 amino acids for PPR and TPR proteins, respectively; and, iv) side chains of amino acids in the central groove are exclusively hydrophilic in PPR proteins whereas they vary considerably in TPR proteins. Another class of PPR proteins is the proteins commonly known as plant combinatorial and modular proteins or PCMPs. They have complex and variable arrangements of PPR motifs in different combinations.^175^,^176^ Apart from the predominant PPR repeat motifs, several other variable motifs have been found at the C-terminus in various PPR proteins. There are three different optional motifs in PPR proteins: E, Eþ, and DYW.^173^ While E and Ep motifs are degenerate, the amino acid sequence of DYW motifs is well conserved, especially Cys and His.^177^ The occurrence of C-terminal motif is optional in classical PPR and has been implicated in the recruitment of catalytic factors for RNA processing.^176^

Most of the known PPR proteins of land plants are nuclear-encoded and targeted to the mitochondria or chloroplasts since they contain a transit peptide at the N-terminus.^175^ Coordination of nuclear and organellar gene expression with organellar functions is essential to maintain cellular homeostasis, and to respond to changes in environmental conditions. Within this context of multiple regulatory signalling pathways, PPR proteins seem to play a significant role.^178^ PPR proteins seem to bind to specific chloroplast transcripts modulating their expression with other general factors.

PPR proteins play essential roles in chloroplast gene expression, affecting transcription RNA processing and stabilization, intron splicing editing and translation (see Table 1 and Fig. 2). To date, only a few PPR proteins affecting chloroplast RNA processing and stabilization have been identified, mostly in Arabidopsis (see Table 1). CRR2 is a member of the plant combinatorial and modular protein (PCMP) family consisting of more than 200 genes in *Arabidopsis*. As mentioned earlier, CRR2 functions in the intergenic processing of chloroplast RNA between *rps7* and *ndhB*, which is possibly essential for *ndhB* translation.^78^ CRP1 is a PPR protein with 14 tandem PPR motifs integrated in a multisubunit protein complex which is necessary for the accumulation of *petB*, *petD*, and *petA* chloroplast mRNAs in maize. The lack of the CRP1 protein results in the loss of the cytochrome b6f complex.^48^,^82^ The CRP1 protein is also directly associated with *petA* and *psaC* mRNAs *in vivo*, activating their translation.^83^ HCF152 is a PPR protein with 12 putative PPR motifs which binds certain areas of the *petB* transcript in *Arabidopsis*. This protein seems to exist in the chloroplast as a homodimer and is not associated with other proteins to form a high molecular mass complex.^79^,^179^,^180^ P67 is another PPR protein that could be involved in chloroplast RNA processing.^80^ Both HCF152 and P67 proteins show a significant similarity to the maize protein CRP1. PGR3 is a protein with 27 PPR motifs which appears to be involved not in the processing but in the stabilization and activation of the *petL* mRNA translation in *Arabidopsis.*^81^ ZmPPR5 is a protein with 8 PPR repeats ortholog of the embryo-essential *Arabidopsis* AtPPR5. This protein specifically binds the trnG-UCC group II intron and stabilizes the *trnG*-UCC precursor by directly protecting an endonuclease-sensitive site. These findings add to the evidence that chloroplast-localized PPR proteins that are embryo essential in *Arabidopsis* function in the biogenesis of the plastid translation apparatus. In rice, OSPPR1 is a protein with 11 PPR repeats involved in the processing of chloroplast transcripts necessary in the early steps of plastid biogenesis.^181^

PPR proteins are also involved in editing of specific chloroplast RNAs. The CRR4 protein belongs to the PCMP protein family with 11 PPR motifs and seems to be essential for RNA editing of *ndhD* in chloroplasts of *Arabidopsis*. It is speculated that CRR4 recognizes the target RNA and facilitates recruitment of general factors for RNA editing events in the chloroplast.^166^ It has been hypothesized that CRR4 protein functions as a trans-acting factor specifically interacting with a target sequence near the *ndhD* editing site, affecting the start codon, and recruiting a putative editing enzyme such as cytidine deaminase, probably via the C-terminal Eþ domain.^119^,^167^ CRR21 is a PPR protein that is involved in the RNA editing of another editing site within *ndhD* transcripts consisting of the conversion of the Ser-128 of NdhD protein to leucine.^168^ *Arabidopsis crr21* mutants are specifically impaired in the RNA editing of this editing site and in the NDH complex suggesting that the Ser128Leu change has important consequences for the function of the NDH complex. Both CRR21 and CRR4 belong to the E+ subgroup of the PLS subfamily that is characterized by the presence of a conserved C-terminal region (the E/E+ domain). This E/E+ domain is highly conserved and exchangeable between CRR21 and CRR4 but it is not essential for RNA binding. Recent investigations suggest that the E/E+ domain has a common function in RNA editing rather than in recognizing specific RNA sequences. CLB19 is a PPR protein similar to the editing specificity factors CRR4 and CRR21, but, unlike them, is implicated in editing of two distinct target sites within the chloroplast, the *rpoA* and *clpP* transcripts. Mutants with a non-functional CLB19 protein show a yellow phenotype with impaired chloroplast development and early seedling lethality. In these mutants, transcript patterns are similar to a defect in the activity of the plastid-encoded RNA polymerase.

PPR proteins are also involved in chloroplast intron splicing. OTP51 is a PPR protein that is required for the splicing of *ycf3* intron 2, and also influences the splicing of several other group-IIa introns. In *Arabidopsis* mutants, the loss of OTP51 has consequences for photosystem-I and photosystem-II assembly, and for the photosynthetic fluorescence characteristics. This protein contains two LAGLIDADG motifs that are found in group-I intron maturases in other organisms. Interestingly, the amino acids reported to be important for maturase activity are conserved whereas amino acids thought to be important for the homing endonuclease activity of other LAGLIDADG proteins are missing in this protein. PPR4 is a chloroplast-targeted protein harbouring both a PPR tract and an RNA recognition motif. The association of PPR4 with the first intron of the plastid *rps12* pre-mRNA and the fact that maize *ppr4* mutants are defective for *rps12* trans-splicing, indicates that this protein is an *rps12* trans-splicing factor.^181^

Thus far, PPRs have been considered exclusively eukaryotic, and they are greatly expanded in plants. However, the factors that underlie the expansion of this gene family in plants are not yet understood. Further studies are necessary to identify the diverse roles of the PPR family of proteins and to understand how PPR proteins help regulate the organellar gene expression and plant development.

## Concluding Remarks

The control of chloroplast gene expression includes several processes that are similar to those of both prokaryotic and eukaryotic systems. These processes are: transcription, RNA processing, translation, and post-translational modifications (Fig. 1). Generally, transcription rates and steady-state mRNA levels are not comparable, suggesting that post-transcriptional RNA processing and stabilization are decisive steps in controlling gene expression in plastids. This step principally includes: RNA cleavage of pre-existing RNAs, RNA stabilization-degradation, intron splicing, and RNA editing. Recently, several nuclear-encoded proteins that participate in chloroplast transcript processing and stabilization have been characterised. Many of them seem to be pentatricopeptide repeat (PPR) proteins implicated in many crucial functions including organelle biogenesis and plant development (Table 1). PPR proteins seem to bind to specific chloroplast transcripts modulating their expression with other general factors and appear to be involved in the control of post-transcriptional gene expression in chloroplasts: in transcript processing, stabilization, editing, and translation. Although it is generally assumed that the PPR motifs form the RNA binding domain, the basis for RNA recognition remains unknown. To add clarity, point mutagenesis and crystal structure analysis studies are needed. Moreover, the identification of interacting enzymes will be crucial to understanding the role of PPR proteins in the editing, splicing, stability and translation of diverse transcripts in chloroplasts. Finally, in spite of the increasing list of PPR proteins, as summarized in Table 1, there is little evidence of their involvement in the regulation of chloroplast metabolism in relation to plant development and in response to environmental changes. To reach this goal, further investigations focused on the behaviour of these newly described proteins in different developmental stages and in response to environmental conditions will be necessary.

## Figures and Tables

**Table 1:** List of pentaricopeptide repeat protein genes involved in the control of chloroplast gene expression.

Organism	Gene	Subfamily	PPR repeats	Target	Possible function	Accession	Reference
***A. thaliana***	**CLB19** (At1g05750)	P-subfamily	9	*rpoA*, *clpP*	Editing of *rpoA* and *clpP* transcripts	Q9MA50	Chateigner-Boutin et al.^169^
	**CRR2** (At3g46790)	PCMP-H	9	*rps7/ndhB*	RNA processing between rps7 and ndhB	NP_190263	Hashimoto et al.^78^
	**CRR4** (At2g45350)	PCMP-E	11	*ndhD*	RNA editing	NP_182060	Kotera et al;^166^ Okuda et al.^167^
	**CRR21** (At5g55740)	P-subfamily	13	*ndhD*	RNA editing	NP_200385	Okuda et al.^168^
	**DG1** (At5g67570)	P-subfamily	7	PEP	Regulation of plastid-encoded polymerase-dependent chloroplast gene expression	NP_201558	Chi et al.^184^
	**HCF152** (At3g09650)	P-subfamily	12	*psbH/petB*	Processing and/or stabilization of polycistronic transcripts of the operon *psbB-psbT-psbH-petB-petD*	NP_187576	Meierhoff et al;^179^ Nakamura et al.^79^
	**OTP51** (At2g15820)	P-subfamily	4	*ycf3*	splicing of *ycf3* intron 2 and other group-IIa introns	NP_565382	de Longevialle et al.^182^
	**P67** (At4g16390)	P-subfamily	2	Not known	Processing or the translation of RNAs	NP_193372	Lahmy et al.^80^
	**PGR3** (At4g31850)	P-subfamily	27	*petL* operon	Stabilization of the primary tricistronic transcript of the *petL* operon	NP_194913	Yamazaki et al.^81^
	**AtPPR4** (At5g04810)	P-subfamily	16	Not known	Ribosome biogenesis	NP_568141	Schmitz-Linneweber et al.^113^
***C. reinhardtii***	**MCA1**	P-subfamily	10	*petA*	Required for stable accumulation of the *petA* transcript	AAK14341	Raynaud et al.^86^
***P. patens***	**PPR531-11**	P-subfamily	11	*rps12, clpP*	Intergenic RNA cleavage between clpP and 5′-*rps12* and the splicing of *clpP* pre-mRNA	BAF02664	Hattori et al.^85^
***O. sativa***	**OsPPR1**	P-subfamily	11	Not known	Chloroplast biogenesis	AAS93059	Gothandam et al.^181^
***Z. mays***	**CRP1**	P-subfamily	14	*petA*, *petD*	Processing of the *petD* and translation of the *petA* and *petD* RNAs	AAC25599	Fisk et al;^82^ Schmitz-Linneweber et al.^83^
	**PPR2**	P-subfamily	11	Not known	Required for plastid ribosome accumulation	AAP37977	Williams and Barkan^183^
	**PPR4**	P-subfamily	16	*rps12*	Trans-splicing of *rps12* RNA and ribosome biogenesis	ABF57644	Schmitz-Linneweber et al.^113^
	**PPR5**	P-subfamily	8	*trnG*-UCC	Stabilization of the *trnG*-UCC tRNA precursor	NP_001106062	Beick et al.^84^
